# Barriers and facilitators to exercise among adult cancer survivors in Singapore

**DOI:** 10.1007/s00520-022-06893-y

**Published:** 2022-02-14

**Authors:** Alexandre Chan, Kayleen Ports, Patricia Neo, Mothi Babu Ramalingam, Ang Tee Lim, Benedict Tan, Nicolas H. Hart, Raymond J. Chan, Kiley Loh

**Affiliations:** 1 Department of Clinical Pharmacy Practice, School of Pharmacy & Pharmaceutical Sciences, University of California, Irvine, USA; 2 Department of Oncology Pharmacy, National Cancer Centre Singapore, Singapore, Singapore; 3 Division of Supportive and Palliative Care, National Cancer Centre Singapore, Singapore, Singapore; 4 Department of Rehabilitation Medicine, Singapore, Singapore; 5 Department of Sport and Exercise Medicine, Changi General Hospital, Singapore, Singapore; 6 Caring Futures Institute, College of Nursing and Health Sciences, Flinders University, Adelaide, South Australia Australia; 7 Cancer and Palliative Care Outcomes Centre, Queensland University of Technology, Brisbane, QLD Australia; 8 Exercise Medicine Research Institute, Edith Cowan University, Joondalup, WA Australia; 9 Institute for Health Research, University of Notre Dame Australia, Fremantle, WA Australia; 10 Division of Medical Oncology, National Cancer Centre Singapore, Singapore, Singapore

**Keywords:** Oncology, Exercise, Cancer survivorship, Counseling, Barriers, Facilitators

## Abstract

**Purpose:**

Exercise can help cancer survivors manage sequela, treatment side effects, improve overall quality of life, and is recommended for most. The purpose of this study was to investigate exercise behavior and factors influencing exercise engagement among cancer survivors at the National Cancer Centre, Singapore (NCCS).

**Methods:**

This cross-sectional study was inclusive of survivors of all cancer types and stages who were at least 21 years of age and had undergone chemotherapy at the NCCS. Surveys were utilized to assess survivor barriers and facilitators to exercise and to retrospectively assess physical activity and exercise behaviors at 4 cancer-related time periods (pre-diagnosis and post-diagnosis before, during, or after chemotherapy).

**Results:**

A total of 102 cancer survivors were enrolled; 60% were diagnosed with stage IV cancer. Predominant cancer types included lower gastrointestinal tract (25.5%) and breast cancer (21.6%). Prior to cancer diagnosis, 90.2% of participants reported aerobic activity satisfying NCCN guidelines. Significant reductions in reported exercise, and physical activity, were observed following cancer diagnosis that persisted during chemotherapy. Key exercise facilitators included the desire to remain healthy (86.3%) and to improve sleep and mental well-being (73.5%). Key barriers included side effects of treatment (52.0%). Only 46.1% of survivors reported receiving exercise guidance from healthcare professionals following diagnosis.

**Conclusion:**

Overall, even among this notably active cohort of Singaporean survivors, opportunities for increased exercise engagement throughout the survivorship continuum remain. Increased education regarding the benefits of exercise to survivors as well as guidance regarding exercise modalities including resistance training is greatly needed as well.

## Introduction

Advancements in cancer detection and treatment have led to decreased cancer mortality rates and a rapidly increasing population of cancer survivors with unique survivorship needs [1]. Cancer survivors, defined by the National Comprehensive Cancer Network (NCCN) as individuals from cancer diagnosis through end of life, often experience reduced quality of life (QoL) due the physiological and psychosocial side effects associated with cancer and its treatments. Prevalent sequelae and side effects include cancer-related fatigue, pain, depression, cognitive difficulties, and sleep disruption that often persist years after treatment completion, in addition to treatment-related comorbidities including cardiovascular disease and type II diabetes [2–4]. The prevalence of serious and often long-term morbidities among survivors, especially those who have undergone chemotherapy, highlights the critical need for effective interventions. One promising intervention of growing interest is exercise.

A growing body of evidence has determined exercise to be a promising intervention for managing the adverse effects of cancer and its treatments, with benefits including improved physical function, cardiorespiratory fitness, cancer-related fatigue, psychosocial well-being, and body composition [5–7]. Further, exercise has been associated with an increased tolerance for cancer medication, reduced risk of cancer recurrence, reduced all-cause mortality, as well as reduced breast, colon, and prostate cancer-specific mortalities [8–10]. Clear and consistent evidence aided in the development of survivor-specific exercise guidelines, the earliest of which recommended at least 150 min of moderate-intensity or 75 min of vigorous-intensity of aerobic exercise per week along with structured resistance training [6, 11, 12]. However, these guidelines have since been updated to be individualized, prescriptive, and less concerned with arbitrary targets [11, 13–15]. Under current guidelines, each individual cancer survivor is recommended to be screened and assessed for sequelae, comorbidities, prior activity levels, and personal goals in order to inform the selection of the appropriate exercise dose, frequency, and modality [6, 11, 13–17].

Despite the abundance of evidence and detailed guidelines, most cancer survivors are not meeting exercise guideline recommendations [4, 12, 18–22]. Preliminary explorations into this phenomena have determined that oncology health care professionals (HCPs) often have limited awareness of exercise guidelines and report feeling underqualified to provide exercise guidance to survivors [13, 18, 22]. However, oncology HCPs have also expressed understanding exercise as an important component of survivorship care, as well as interest in receiving further education and multidisciplinary team support to address this cancer survivorship need [13, 18]. To date, preliminary investigations into facilitators and barriers of exercise for cancer survivors have been conducted in cohorts from countries including the USA, Australia, Norway, Korea, Canada, and the UK [20, 22–25] that are predominantly western civilizations with the exception of Korea. Survivor-specific barriers to exercise identified among these cohorts have included lack of time, fatigue, treatment-related side effects, and lack of education regarding exercise recommendations and benefits [13, 18, 22]. Key facilitators have included the ability to regain an aspect of control over one’s health and mental wellbeing [22]. However, culture has a profound influence on health behaviors and existing findings may have limited generalizability to cancer survivors in Singapore.

Cancer survivorship care in Singapore is in its early stages [26]. Historically, Singapore’s approach to cancer survivorship has been surveillance-focused and oncologist-centric, in contrast to the shared-care models of survivorship care often seen in North American and European countries [26, 27]. With a rising prevalence of survivors and recognition of structural changes needed to meet growing national survivorship needs, Singapore held its first cancer supportive and survivorship care forum in December of 2016. Several key principles for the nationwide improvement of cancer survivorship care were identified including the necessity of a survivor-centered focus, integrated and coordinated care, and a strong research infrastructure for the development of evidence-based programs [26]. In accordance with forum findings, the National Cancer Center, Singapore (NCCS) began the development of a new center dedicated to meeting the needs of Singapore’s constantly growing cancer survivor population. A key area of interest for the new center is the implementation of evidence-based interventions to manage poorly understood survivorship issues, including standardized and structured exercise programs. However, there is a dearth of research regarding survivor exercise engagement and factors influencing engagement in Singapore that would be critical to the development and implementation of an effective exercise program for this unique population. Therefore, this study was broadly designed to (1) investigate barriers and facilitators to exercise among Singaporean cancer survivors who are undergoing, or have undergone, chemotherapy at the NCCS and to (2) assess Singaporean cancer survivors’ exercise behaviors across the survivorship continuum.

## Methods

### Study design

This cross-sectional study was conducted at the NCCS between August and October 2019. The NCCS is the largest ambulatory cancer center in Singapore, treating up to 70% of all adult cancer patients. Ethics approval was granted by SingHealth Centralised Institutional Review Board (CIRB Ref: 2019/2528) prior to study commencement.

### Inclusion and exclusion criteria

This study recruited cancer survivors who were at least 21 years old, able to read and understand English, diagnosed with any cancer of any stage (I–IV) by an oncologist, and who had received chemotherapy at the NCCS within the past 12 months prior to study enrollment. Cancer survivors were excluded if they had cognitive or severe psychiatric disorders that investigators judged to likely impair their ability to provide informed consent or answer questionnaires.

### Study procedures

Survivors at the NCCS were identified by their oncologist, approached during either a routine chemotherapy session or consultation visit and screened for eligibility. Survivors who consented to participate were given a set of self-administered survey questionnaires in English which were collected upon completion. Each set of questionnaires took approximately 20–30 min to complete.

### Measures

#### Demographic and clinical information

Demographic information (age, gender, height and weight, ethnicity, education, marital status, and employment status) and clinical information (cancer type, stage of cancer, Eastern Cooperative Oncology Group (ECOG) performance status, comorbidities, cancer treatments received, and time since chemotherapy completion) were collected through self-administered questionnaire.

#### Exercise and physical activity behaviors

Exercise and physical activity behavior were assessed through an investigator-designed recall questionnaire. Survivors were asked to classify weekly activity into two categories: exercise or physical activity. Exercise was defined as purposeful, prescriptive, programmed, and progressive activities of a specific nature [28]. Physical activity and exercise were further categorized into 3 levels of intensity: light (no noticeable change in breathing pattern), moderate (breath quickens but not out of breath, develop light sweat after approximately 10 min, can talk but cannot sing), and vigorous (breathing is deep and rapid, develop sweat after several minutes, cannot say a few words without stopping to catch a breath) [11]. Survivors were asked to recall their exercise and physical activity behavior at each intensity level across four different cancer-related time periods: (1) pre-diagnosis, (2) post-diagnosis before chemotherapy, (3) during chemotherapy, and (4) post-chemotherapy. Post-chemotherapy activity was reported only by participants who had completed chemotherapy at the time of study participation. Information on activity type (walking, cycling, housework, etc.) and estimated weekly duration was collected in free-form text.

#### Perceived exercise barriers and facilitators

Cancer survivors’ perceived barriers and facilitators to exercise were assessed using an investigator-designed questionnaire containing 20 barriers and 15 facilitators selected a priori based on findings from existing literature [20, 22–25, 29]. Participants were asked to select each facilitator and barrier they believed influenced their exercise behavior.

#### Exercise guidance and education

**Fig. 1:**
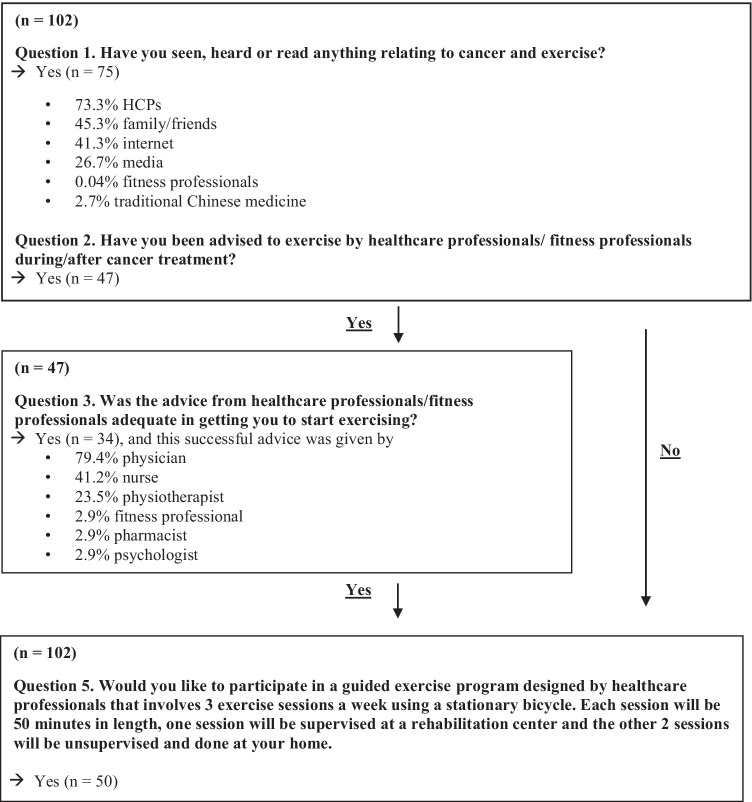
Survivor exercise guidance and education survey

History of exercise education and guidance provision was assessed using 5 conditional, closed-ended questions (Fig. 1). Participants were asked whether they had previous exposure to information regarding exercise and cancer, were advised to exercise by a HCP (e.g., oncologist, physiotherapist, social worker) or fitness professional (e.g., personal trainer, instructor) following cancer diagnosis, and whether this information had been adequate to motivate them to initiate exercise. Finally, participant interest in a guided exercise program designed by study investigators for potential implementation at the new NCCS facility was assessed. This program was described as 3, 50-min sessions per week on a stationary bicycle: one supervised at a rehabilitation center and 2 unsupervised at home.

### Statistical analysis

Statistical analyses were performed in SAS 9.3 (SAS Institute, Cary, NC). Descriptive statistics were used to describe demographic, clinical, and survey data. Categorical variables were presented as proportions and continuous variables were summarized as mean, standard deviation (SD), and range. For the purposes of data analysis, participants were divided into two subgroups: (1) those who were undergoing chemotherapy at the time of participation and (2) those who had completed therapy treatment at the time of participation. Differences between subgroups were assessed using chi-square tests for categorical demographic and clinical variables. Fisher’s exact tests were used when cell counts were below 5. Independent *t*-tests were used to compare age and body mass index (BMI) following confirmation of normality through QQ-plots. Differences between subgroups in the proportion of participants reporting each individual facilitator and barrier were compared using chi-square tests. Self-reported weekly duration of physical activity and exercise were summarized as median and interquartile range (IQR). Wilcoxon signed-rank test was used to test for differences in activity levels at each timepoint compared to activity levels prior to cancer diagnosis. Additionally, the proportion of respondents meeting NCCN cancer survivorship aerobic exercise guidelines at each time period was assessed. McNemar’s test for paired samples was utilized to compare the proportion of participants meeting guidelines at each timepoint to the proportion meeting guidelines prior to cancer diagnosis. A *p* value less than 0.05 was considered statistically significant.

## Results

### Demographic and clinical characteristics

A total of 221 survivors were identified and approached for participation in this study. Of the 221, 202 were confirmed to meet eligibility criteria and 102 (50.5%) consented to participate. Of the 102 study participants, 65 (63.7%) were undergoing chemotherapy at the time participation and 37 (36.3%) had completed chemotherapy. Participants were primarily Chinese (84.3%), male (52.9%), married (72.5%), graduates/post-graduates (38.2%), and not working at the time of participation (43.1%) (Table 1). The mean (± SD) age of participants was 54.6 ± 12.7 and the mean (± SD) BMI was 23.1 ± 4.0. Thirty-nine respondents (38.2%) reported additional comorbidities, including hypertension (17.6%), diabetes (15.7%), and high cholesterol (13.7%). No significant differences in demographic characteristics between participants undergoing chemotherapy at the time of participation and those who had completed chemotherapy were observed.

**Table 1:** Demographic and clinical characteristics of study participants

	Undergoing chemotherapy (*N* = 65)	Completed chemotherapy (*N* = 37)	All survivors (*N* = 102) *N* (%)	*p* value^a^
Demographic characteristics				
Gender				0.512
Male	36 (55.4)	18 (48.6)	54 (52.9)	
Female	29 (44.6)	19 (51.4)	48 (47.1)	
Race				0.875
Chinese	53 (81.5)	33 (89.2)	86 (84.3)	
Malay	6 (9.2)	2 (5.4)	8 (7.8)	
Indian	4 (6.2)	1 (2.7)	5 (4.9)	
Other	2 (3.1)	1 (2.7)	3 (2.9)	
Marital status				0.926
Married	48 (73.8)	26 (70.3)	74 (72.5)	
Single	11 (16.9)	8 (21.6)	19 (18.6)	
Divorced	5 (7.7)	3 (8.1)	8 (7.8)	
Widowed	1 (1.5)	0 (0.0)	1 (1.0)	
Living alone				0.250
No	58 (89.2)	30 (81.1)	88 (86.3)	
Yes	7 (10.8)	7 (18.9)	14 (13.7)	
Education level				0.699
Primary	13 (12.7)	4 (10.8)	9 (13.8)	
Secondary	30 (29.4)	10 (27.0)	20 (30.8)	
Pre-university	20 (19.6)	6 (16.2)	14 (21.5)	
Graduate/post-graduate	39 (38.2)	17 (45.9)	22 (33.8)	
Employment status				0.173
Not working	44 (43.1)	14 (37.8)	44 (43.1)	
Full-time employment	42 (41.2)	18 (48.6)	42 (41.2)	
Part-time employment	9 (8.8)	1 (2.7)	9 (8.8)	
Self-employed	7 (6.9)	4 (10.8)	7 (6.9)	
**Age** (years): mean ± SD (range)	54.0 ± 12.6 (21–86)	55.8 ± 12.9 (31–84)	54.6 ± 12.7 (21–86)	0.493
Clinical characteristics				
Cancer type				0.036*
Lower gastrointestinal tract	19 (29.2)	7 (18.9)	26 (25.5)	
Breast	15 (23.1)	7 (18.9)	22 (21.6)	
Hematologic malignancies	2 (3.1)	7 (18.9)	9 (8.8)	
Head and neck	9 (13.8)	0 (0.0)	9 (8.8)	
Female reproductive organs	4 (6.2)	4 (10.8)	8 (7.8)	
Hepatobiliary system	5 (7.7)	3 (8.1)	8 (7.8)	
Upper gastrointestinal tract	4 (6.2)	3 (8.1)	7 (6.9)	
Thorax	4 (6.2)	2 (5.4)	6 (5.9)	
Genitourinary cancers	1 (1.5)	3 (8.1)	4 (3.9)	
Soft tissue sarcoma	2 (3.1)	1 (2.7)	3 (2.9)	
Cancer stage				0.059
I	4 (6.2)	6 (16.2)	10 (9.8)	
II	6 (9.2)	6 (16.2)	12 (11.8)	
III	12 (18.5)	9 (24.3)	21 (20.6)	
IV	43 (66.2)	15 (40.5)	58 (56.9)	
Not applicable^b^	0 (0.0)	1 (2.7)	1 (1.0)	
ECOG performance status^c^				0.857
0	27 (41.5)	17 (45.9)	44 (43.1)	
1	36 (55.4)	19 (51.4)	55 (53.9)	
2	2 (3.1)	1 (2.7)	3 (2.9)	
Cancer treatments received				
Chemotherapy	65 (100.0)	37 (100.0)	102 (100.0)	-
Surgery	35 (53.8)	27 (73.0)	62 (60.8)	0.057
Radiation	15 (23.1)	8 (21.6)	23 (22.5)	0.866
Targeted	9 (13.8)	8 (21.6)	17 (16.7)	0.311
Hormonal	3 (4.6)	6 (16.2)	9 (8.8)	0.069
Comorbidities				
Hypertension	11 (16.9)	7 (18.9)	18 (17.6)	0.799
Diabetes	11 (16.9)	5 (13.5)	16 (15.7)	0.649
High cholesterol	8 (12.3)	6 (16.2)	14 (13.7)	0.581
Liver disease	3 (4.6)	0 (0.0)	3 (2.9)	0.552
Osteoporosis	2 (3.1)	2 (5.4)	4 (3.9)	0.620
Cardiovascular disease	1 (1.5)	2 (5.4)	3 (2.9)	0.297
Glaucoma	1 (1.5)	0 (0.0)	1 (1.0)	1.000
Lung disease	1 (1.5)	0 (0.0)	1 (1.0)	1.000
Arthritis	1 (1.5)	0 (0.0)	1 (1.0)	1.000
Kidney disease	1 (1.5)	0 (0.0)	1 (1.0)	1.000
Others	3 (4.6)	4 (10.8)	7 (6.9)^d^	0.665
**BMI:** mean ± SD (range)	23.0 ± 4.4	23.5 ± 3.3	23.1 ± 4.0 (13.1–35.7)	0.484
**Time since chemotherapy completion** (*N* = 37): mean ± SD (range)	–	4.6 ± 3.9 (0–12)	4.6 ± 3.9 (0–12)	–

^a^*χ*^2^ test was used to test for associations between chemotherapy completion and categorical variables, Fisher’s exact tests were used when cell counts < 5, and independent *t*-tests were used to test for associations between chemotherapy completion and the means of continuous variables

^b^Cancer staging is unavailable for acute myeloid leukemia

^c^ECOG 0 = fully active, able to carry on all pre-disease performance without restriction; ECOG 1 = restricted in physically strenuous activity but ambulatory and able to carry out work of a light or sedentary nature, e.g., light housework and office work; ECOG 2 = ambulatory and capable of all self-care but unable to carry out any work activities; up and about more than 50% of waking hours

^d^Endometriosis (*n* = 1), dermatomyositis (*n* = 1), age-related macular degeneration on left eye (*n* = 1), myasthenia gravis (*n* = 1), thyroid (*n* = 1), psoriasis (*n* = 1), and PCOS (*n* = 1)

^*^p value < 0.05

Participants were primarily diagnosed with stage IV cancer (60%). The most prevalent cancer types included lower gastrointestinal tract (25.5%) and breast cancer (21.6%). Participants undergoing chemotherapy at the time of study participation had significantly higher proportions of gastrointestinal tract and head and neck cancer, whereas participants who had completed chemotherapy had higher proportions of hematologic malignancies and genitourinary cancers. All participants received chemotherapy treatment, 60.8% had cancer-related surgery, and 22.5% received radiation therapy. Regarding disease impact, 43.1% of participants were fully active and able to continue pre-disease activity without restriction (ECOG score of 0), whereas 53.9% faced restrictions in physically strenuous activities but remained ambulatory (ECOG score of 1). Among the 37 participants who had completed chemotherapy at the time of study participation, the mean (± SD) time since chemotherapy completion was 4.6 ± 3.9 months.

### Exercise behavior

Prior to cancer diagnosis, 90.2% of all study participants reported physical activity and exercise satisfying NCCN cancer survivorship aerobic exercise guidelines (Table 2). This proportion was significantly reduced following cancer diagnosis (69.6%, *p* < 0.001) and remained reduced during chemotherapy (65.7%, *p* < 0.001). Among the 37 individuals who had completed chemotherapy, 75.7% met exercise guidelines after chemotherapy completion, which was not significantly different than the proportion in that subset who met guidelines prior to cancer diagnosis (83.8%, *p* = 0.180).

**Table 2:** Proportion of respondents meeting NCCN cancer survivorship aerobic exercise guidelines^a^ across 4 cancer-related time periods

Time period	All respondents (*N* = 102) *N* (%)	*p* value^b^	Undergoing chemotherapy (*n* = 65)	*p* value^b^	Completed chemotherapy (*N* = 37) *N* (%^b^)	*p* value^b^
Pre-diagnosis	92 (90.2)	Ref	61 (93.9)	Ref	31 (83.8)	Ref
Post-diagnosis, before chemotherapy	71 (69.6)	< 0.001	49 (75.4)	0.001	22 (59.5)	0.003
During chemotherapy	67 (65.7)	< 0.001	46 (70.8)	< 0.001	21 (56.8)	0.002
Post-chemotherapy^c^	–	–	–	–	28 (75.68)	0.180

^a^Meeting the aerobic activity guideline is defined as at least 150 min of moderate-intensity or 75 min of vigorous physical activity and/or exercise per week

^b^McNemar’s test for paired samples was utilized to compare proportion of participants meeting activity guidelines at each timepoint to proportion at pre-diagnosis

^c^Post-chemotherapy proportions only include respondents who have completed chemotherapy (*N* = 37)

Moderate-intensity aerobic exercise was the most reported across all time periods. The median (IQR) weekly duration of moderate-intensity aerobic exercise across all participants decreased from 60 (0 to 157) min/week prior to cancer diagnosis to 0 (0 to 105) min/week following diagnosis (*p* < 0.001) and remained significantly lower during chemotherapy (*p* < 0.001) (Table 3). However, among participants who had completed chemotherapy, the median weekly duration of moderate-intensity aerobic exercise after chemotherapy completion was not significantly different than prior to diagnosis (*p* = 0.297). The same pattern was observed for vigorous-intensity aerobic exercise, the median (IQR) weekly duration decreased from 0 (0 to 25) min/week prior to cancer diagnosis to 0 (0 to 0) min/week following diagnosis (*p* < 0.001), and the median weekly duration after chemotherapy completion was not significantly different than prior to diagnosis (*p* = 0.539). Cancer diagnosis was also associated with statistically significant decreases in moderate-intensity physical activity; the median (IQR) decreased from 420 (140.0–840.0) to 221 (52.5–570.0) min/week (*p* < 0.001). This decrease was sustained during chemotherapy (*p* = 0.010) and, among the subset who had completed chemotherapy, after chemotherapy completion as well (*p* = 0.017). Across all time periods, light and moderate physical activity were more commonly reported than light and moderate exercise. Walking was the most common exercise modality reported across the cancer-related time periods (Table 4).

**Table 3:** Median (IQR) reported weekly physical activity and exercise durations of participants by intensity level, across 4 cancer-related time periods (*N* = 102)

Intensity	Pre-diagnosis		Post-diagnosis, before chemotherapy		During chemotherapy		Post-chemotherapy (*N* = 37)^b^	
	Duration—min/week, median (IQR)	*p* value^a^	Duration—min/week, median (IQR)	*p* value^a^	Duration—min/week, median (IQR)	*p* value^a^	Duration—min/week, median (IQR)	*p* value^a^
Exercise								
Light	0.0 (0.0–0.0)	Ref	0.0 (0.0–0.0)	0.502	0 (0.0–20.0)	0.947	0.0 (0.0–20.0)	0.275
Moderate	60.0 (0.0–157.5)	Ref	0.0 (0.0–105.0)	< 0.001*	0.0 (0.0–140.0)	0.010*	120.0 (25.0–187.5)	0.297
Vigorous	0.0 (0.0–25.0)	Ref	0.0 (0.0–0.0)	< 0.001*	0.0 (0.0–0.0)	< 0.001*	0.0 (0.0–0.0)	0.539
Physical activity								
Light	0.0 (0.0–50.0)	Ref	0.0 (0.0–105.0)	0.445	0.0 (0.0–87.5)	0.321	0.0 (0.0–100.0)	0.953
Moderate	420.0 (140.0–840.0)	Ref	221.3 (52.5–570.0)	< 0.001*	180.0 (30.0–540.0)	0.010*	220.0 (20.0–455.0)	0.017*
Vigorous	0.0 (0.0–0.0)	Ref	0.0 (0.0–0.0)	1.000	0.0 (0.0–0.0)	1.000	0.0 (0.0–0.0)	1.000

^a^*p* values based on Wilcoxon signed rank test with pre-diagnosis value for each intensity level as the reference group

^b^Post-chemotherapy data is only available for participants who completed chemotherapy at the time of study participation (*N* = 37)

^*^p value < 0.05

**Table 4:** Moderate and vigorous exercise modalities reported by study participants as free-text across 3 cancer-related time periods (*N* = 102)

Before diagnosis		After diagnosis, before treatment		During chemotherapy	
Activity type	*N* (%)	Activity type	*N* (%)	Activity type	*N* (%)
Moderate exercise types reported					
Walk	37 (36.3)	Walk	29 (28.4)	Walk	35 (34.3)
Jog/run	15 (14.7)	Jog/run	4 (3.9)	Jog/run	2 (2.0)
Cycle	6 (5.9)	Cycle	3 (2.9)	Cycle	2 (2.0)
Gym/physical training	6 (5.9)	Gym/physical training	2 (2.0)	Gym/physical training	3 (2.9)
Sports	4 (3.9)	Sports	1 (1.0)	Sports	1 (1.0)
Aerobics (unspecified)	3 (2.9)	Aerobics (unspecified)	2 (2.0)	Aerobics (unspecified)	4 (3.9)
Swim	3 (2.9)	Swim	0 (0.0)	Swim	0 (0.0)
Yoga	2 (2.0)	Yoga	0 (0.0)	Yoga	0 (0.0)
Other^a^	4 (3.9)	Other	2 (2.0)	Other	0 (0.0)
Vigorous exercise types reported					
Gym/physical training	11 (10.8)	Gym/physical Training	3 (2.9)	Gym/physical training	0 (0.0)
Jog/run	7 (6.9)	Jog/run	1 (1.0)	Jog/run	1 (1.0)
Swim	9 (8.8)	Swim	1 (1.0)	Swim	1 (1.0)
Other^b^	6 (5.9)	Other	3 (2.9)	Other	2 (2.0)

^a^Includes pilates (*n* = 1), trek (*n* = 1), calisthenics (*n* = 1), and Zumba (*n* = 1)

^b^Includes basketball (*n* = 1), boxing (*n* = 1), calisthenics (*n* = 1), taekwondo (*n* = 1), trek (*n* = 1), and mountain biking (*n* = 1)

### Perceived exercise barriers and facilitators

The most commonly reported barriers to exercise include adverse effects from treatment (52.0%), lack of self-discipline (32.4%), weather (43.1%), and other health issues (27.5%) (Table 5). The most commonly reported facilitators to exercise include the desire to remain healthy and productive (84.3%), to improve sleep and mental well-being (72.5%), encouraged by family and friends (52.0%), having exercised prior to treatment with a desire to maintain this activity (50%), increase confidence (50%), help cope better with cancer treatment and side effects of the treatment (48%), and to improve independence and self-control (47.1%) (Table 5). No significant differences in facilitators or barriers were observed between participants undergoing chemotherapy and those who had completed chemotherapy.

**Table 5:** Perceived facilitators and barriers to exercise among study participants

Facilitators	All survivors (*N* = 102) *N* (%)	Undergoing chemotherapy (*n* = 65) *N* (%)	Completed chemotherapy (*n* = 37) *N* (%)	*p* value^a^
Personal				
Remain healthy and productive	86 (84.3)	57 (87.7)	29 (78.4)	0.261
Improve sleep and mental well-being	74 (72.5)	50 (76.9)	24 (64.9)	0.249
Exercised prior to treatment with a desire to maintain this activity	51 (50.0)	31 (47.7)	20 (54.1)	0.681
Increase confidence	51 (50.0)	30 (46.2)	21 (56.8)	0.410
Cope better with cancer treatment and side effects from treatment	49 (48.0)	33 (50.8)	16 (43.2)	0.539
Improve independence and self-control	48 (47.1)	33 (50.8)	15 (40.5)	0.410
Reduce adverse effects from treatment	45 (44.1)	29 (44.6)	16 (43.2)	1.000
Positive experience with exercise prior to treatment	45 (44.1)	28 (43.1)	17 (45.9)	0.837
Prevent cancer recurrence	40 (39.2)	22 (33.8)	18 (48.6)	0.205
Encouragement	36 (35.3)	21 (32.3)	15 (40.5)	0.518
Sufficient time	33 (32.4)	25 (38.5)	8 (21.6)	0.123
Social				
Interactions with other cancer patients through exercise programs	34 (33.3)	21 (32.3)	13 (35.1)	0.829
Environmental				
Encouraged by family and friends to exercise	53 (52.0)	32 (49.2)	21 (56.8)	0.539
Encouraged by healthcare professionals to exercise	45 (44.1)	25 (38.5)	20 (54.1)	0.150
Accessible programs tailored to cancer patients	37 (36.3)	23 (35.4)	14 (37.8)	0.833
Barriers	All survivors (*N* = 102) *N* (%)	Undergoing chemotherapy (*N* = 65)	Completed chemotherapy (*n* = 37)	*p* value^a^
Personal				
Adverse effects from treatment (e.g., lack of energy, fatigue, numbness, tingling, muscle weakness, pain, depression, anxiety, limited joint movement, vomiting)	53 (52.0)	30 (46.2)	23 (62.2)	0.150
Lack of self-discipline	33 (32.4)	19 (29.2)	14 (37.8)	0.387
Exercise limited by other health issues	28 (27.5)	17 (26.2)	11 (29.7)	0.818
Fear of injury	22 (21.6)	15 (23.1)	7 (18.9)	0.803
Lack of time	21 (20.6)	17 (26.2)	4 (10.8)	0.078
Exercise is not a priority (e.g., work/family responsibilities)	20 (19.6)	15 (23.1)	5 (13.5)	0.305
Lack of interest in exercise	18 (17.6)	9 (13.8)	9 (24.3)	0.279
Exercise is not in routine	17 (16.7)	12 (18.5)	5 (13.5)	0.591
Inconvenient exercise schedule	11 (10.8)	9 (13.8)	2 (5.4)	0.320
Uncertainty in use of fitness equipment and type of appropriate exercises	10 (9.8)	6 (9.2)	4 (10.8)	1.000
Unawareness of the need to exercise	6 (5.9)	6 (9.2)	0 (0.0)	0.084
Exercise will make the cancer progress further	2 (2.0)	2 (3.1)	0 (0.0)	0.533
Social				
Lack of company	23 (22.5)	12 (18.5)	11 (29.7)	0.222
Environmental				
Weather (e.g., wet, warm, windy)	44 (43.1)	27 (41.5)	17 (45.9)	0.683
Cost of exercising	16 (15.7)	12 (18.5)	4 (10.8)	0.401
Lack of access to training facility or equipment	11 (10.8)	6 (9.2)	5 (13.5)	0.522
Lack of appropriate exercise facility	10 (9.8)	6 (9.2)	4 (10.8)	1.000
Lack of knowledgeable exercise staff	10 (9.8)	6 (9.2)	4 (10.8)	1.000
Warned by healthcare professionals not to exercise	7 (6.9)	5 (7.7)	2 (5.4)	1.000
Warned by family/friends not to exercise	4 (3.9)	3 (4.6)	1 (2.7)	1.000

^a^*χ*^2^ test was used to test for associations between chemotherapy completion and barriers; Fisher’s exact test was utilized when cell counts < 5

### Exercise guidance and education

Most respondents reported receiving information about cancer and exercise (73.5%; *n* = 75/102), primarily from HCPs (e.g., oncologists, physiotherapists, social workers) (73.3%; *n* = 55/75) (Fig. 1). Of the 47 participants (46.1%; *n* = 47/102) who reported having been advised to perform exercise by healthcare or fitness professionals following cancer diagnosis, all reported they believed exercise would be beneficial to their health and wellbeing prior to receiving guidance. Thirty-four of those participants (72.3%; *n* = 34/47) reported the advice received had been adequate in getting them to start exercising. Fifty participants (49%; *n* = 50/102) expressed interest in the proposed investigator-designed exercise program.

## Discussion

Physical activity and exercise behavior of cancer survivors at NCCS were examined across the continuum of survivorship, and perceived barriers and facilitators to exercise (i.e., purposeful, prescriptive, programmed, and progressive activities targeting various bodily systems [28]) were described. Surprisingly, a large proportion of survivors in this cohort met the aerobic exercise NCCN guidelines prior to cancer diagnosis. This finding is distinct from observations among previous survivor populations in the USA, Canada, Germany, and Korea [4, 19, 21, 30–32], and is reflective of the generally healthy BMIs reported in this cohort, as well as the high levels of societal physical activity described in Singapore’s National Health Survey (NHS). Limited existing investigations into patterns of physical activity in Singapore have shown travel-related activity to be a large contributor to physical activity, which could be related to Singapore’s unique public transportation infrastructure and high taxes on private car ownership [33]. Cultural differences may also have influenced the comparatively high physical activity levels observed in this Singapore survivor cohort; however, further research is still greatly needed. In line with previous investigations [21, 30, 31, 34–36], the point of diagnosis remained a marker for the significant decline in both incidental physical activity and purposeful exercise. Therefore, even among this notably active cohort of survivors, opportunities for increased engagement in exercise throughout the survivorship continuum remain. Additionally, the point of diagnosis could serve as a key moment for the initiation of discussion regarding exercise.

As seen in previous investigations, the most commonly reported barrier to exercise was adverse effects from treatment, which encompassed a range of symptoms such as fatigue, muscle weakness, and pain (Table 5) [22, 37]. However, despite guidelines recommending the utilization of exercise to reduce cancer treatment-related sequelae [5–7], less than half of participants reported reductions in adverse events from treatment as a facilitator of exercise. These findings suggest a lack of education among Singaporean cancer survivors regarding the benefits of exercise that has been similarly observed in international investigations [22, 37]. This is further supported by the finding that less than half of participants reported having been directly advised to engage in exercise following their cancer diagnosis. Therefore, the effective implementation of an exercise intervention at the NCCS for cancer survivors will require evidence-based educational components to motivate and guide engagement. Optimistically, participant survey responses were generally reflective of positive perceptions of exercise and a receptiveness to exercise guidance. Less than one-fifth of participants reported a lack of interest in exercise and exercise not being a priority as barriers to exercise engagement, and the majority of participants reported facilitators such as the desire to remain healthy and productive and previous participation in exercise as facilitators. Additionally, contrary to previous investigations [22], only 20.6% of participants noted a lack of time as a barrier, suggesting a previously identified key barrier to engagement may be less prominent among this cohort. Further, 72% (34/47) of participants who reported having been advised to exercise after cancer diagnosis reported that the advice was adequate in getting them to initiate exercise. Overall, these findings are supportive of the positive reception of exercise education and an appropriately designed and promoted exercise intervention for survivors at the NCCS.

In line with previous studies, walking was the most reported activity among participants [22–24, 38]. Although walking is an aerobic exercise activity, to maximize the benefits of exercise among cancer survivors and meet all components of the NCCN exercise guidelines, Singaporean cancer survivors must prioritize participation in resistance training activities. Resistance training, defined as muscle strengthening and muscle building exercises, is key for addressing important cancer-related side effects including bone and muscle loss, fragility, loss of physical function, and fall prevention, especially among metastatic cancer survivors who compose two-thirds of this study population [12, 13, 39–41]. Survivors with metastases have previously reported hesitancy to conduct exercise, particularly resistance training, without supervision due to issues including fear of fracture, bone metastases, and fragility [6, 7, 15, 42–44]. With 21.6% of cancer survivors reporting fear of injury as a barrier to exercise and an overwhelming majority engaging primarily in walking, individualized guidance and education regarding the appropriate and safe engagement in resistance training will be needed at the NCCS to optimize the benefits of exercise among survivors.

Although the necessity of survivor education regarding the benefits of exercise has been highlighted in this investigation, significant barriers to oncologist exercise promotion have been identified in literature that will require consideration during implementation at the NCCS. Oncologist lack of time and lack of knowledge regarding exercise and cancer survivorship [18] will likely be influential in Singapore where the oncologist-centric model of cancer care has historically placed the burden of addressing complex survivorship needs on the oncologists [13, 18, 26]. Therefore, not only is oncologist education regarding exercise guidelines for cancer survivors recommended, but multidisciplinary team support would be as well. Pilot studies examining the feasibility and acceptability of survivor-specific exercise intervention programs in other international cohorts have seen low referral and participation rates [40, 41, 45]. Filling the research-to-practice gap in Singapore exercise oncology and successfully integrating exercise into standard oncology care will further require the development of intentionally designed, standardized care pathways and implementation plans involving multidisciplinary team support [40, 41, 45].

The findings of this investigation should be interpreted with cautions. Physical activity and exercise are self-reported and subject to recall bias. Additionally, variability in the ability to recall based on the time since completion of each survivorship stage is likely reflected in these findings. The survey tool utilized to capture physical activity and exercise data was an investigator-developed, non-validated tool and thus not confirmed to be psychometrically sound. Additionally, given that resistance training is often poorly understood by cancer survivors and difficult to accurately capture through self-reported survey, only the formal assessment of aerobic exercise NCCN guideline adherence, and not resistance training guideline adherence, was conducted. However, all exercise types engaged were collected in free-form text as seen in Table 4. Further, the findings of this study did not control for the recency of treatments including surgery and radiation, which would likely influence reported exercise behavior. Additionally, given that only 50.5% of approached patients consented to participate in the study, the findings could be susceptible to sampling bias. Finally, participants were largely active prior to cancer diagnosis, viewed exercise in a positive light, were diagnosed with stage IV cancer, and therefore the findings should be interpreted within that context.

## Conclusion

This is the first study investigating exercise activity, barriers, and facilitators among cancer survivors at NCCS, which is the largest ambulatory cancer center in Singapore. This study outlined necessity of increased exercise engagement following cancer diagnosis, the necessity of increased exercise education among survivors, outlined barriers and facilitators to exercise engagement, and revealed walking as the primarily exercise modality among survivors. The findings of this study provide vital preliminary data that can serve both in supporting and in informing the design of survivor-specific exercise interventions that can be integrated into Singapore’s health care infrastructure at the NCCS in order to maximize the benefits of exercise among survivors.

## Data Availability

Data could be provided upon request.
